# Optimizing corn silage quality during hot summer conditions of the tropics: investigating the effect of additives on *in-silo* fermentation characteristics, nutrient profiles, digestibility and post-ensiling stability

**DOI:** 10.3389/fpls.2023.1305999

**Published:** 2023-11-23

**Authors:** Nazir Ahmad Khan, Nadar Khan, Shaoxun Tang, Zhiliang Tan

**Affiliations:** ^1^ Key Laboratory for Agro-Ecological Processes in Subtropical Region, National Engineering Laboratory for Pollution Control and Waste Utilization in Livestock and Poultry Production, Hunan Provincial Key Laboratory of Animal Nutritional Physiology and Metabolic Process, Institute of Subtropical Agriculture, The Chinese Academy of Sciences, Changsha, Hunan, China; ^2^ Department of Animal Nutrition, The University of Agriculture, Peshawar, KP, Pakistan

**Keywords:** silage additives, homofermentative, heterofermentative, corn silage, in silo fermentation quality, CNCPS subfraction, microbial count, aerobic stability

## Abstract

Corn crop grown and ensiled at high temperature have lower water soluble carbohydrates (WSC), epiphytic lactic acid bacteria (LAB) population, lactic acid concentration, fermentation quality and aerobic stability. This study systematically investigated the effects of heterofermentative LAB (hetLAB), homofermentative LAB (homLAB), molasses and their mixture (MIX) on *in-silo* fermentation characteristics, chemical profiles, Cornell Net Carbohydrate and Protein System (CNCPS) carbohydrate subfractions, *in vitro* digestibility (DMD), microbial count, and post-ensiling aerobic stability of whole crop corn silage during hot summer (30 to 45°C) condition. Corn hybrids (P30K08 and DK6789) were ensiled at targeted dry matter (DM) of 330 g/kg for 0, 3, 7, 21, and 150 days in 3 L silos, without additive (CCS) or treated with hetLAB (4×10^6^ cfu/g *Lactobacillus buchneri*), homLAB (1×10^6^ cfu/g of *L. plantarum*), molasses (3% of fresh forage) or MIX (half of individual doses of homLAB, hetLAB and molasses) additives. The CCS, homLAB, hetLAB, molasses, or MIX treated chopped material of each hybrid were ensiled in 16 replicate silos at a density of 260 kg of DM/m^3^. Compared to CCS, the additives significantly improved silage nutritional and fermentation quality, DM digestibility (*in vitro*), count of LAB, DM recovery and aerobic stability, and decreased counts of yeast and mold. Among the inoculants, the homLAB and MIX inoculated silages had greatest improvement in fermentation quality and nutritional value. The homLAB produced corn silage with the highest (*P* < 0.05) content of lactic acid, and soluble carbohydrates, and lowest contents of acetic acid, NH_3_-N and pH, demonstrating desirable and restricted *in silo* fermentation. On the other hand, the hetLAB inoculated silages had the greatest (*P* < 0.05) value of acetic acids, highlighting greater aerobic stability. Interestingly, the MIX silages followed the hetLAB in acetic acid value and homLAB in lactic acid value. Notably, without additive stable pH was not achieved during 21 days, with application of molasses, hetLAB and the MIX inoculants stable pH was achieved during 7 days, and with homLAB stable pH was achieved during the first 3 days of ensiling. The greatest numbers of viable LAB were recorded in homLAB (8.13 log cfu/g) and MIX (7.89 log cfu/g) inoculated silages, while the lowest for CCS (6.29 log cfu/g). The lowest yeast (1.48 log cfu/g) and mold (0.22 log cfu/g) were recorded for hetLAB inoculated silage. The greatest (*P* < 0.05) DM recovery was recorded for hetLAB (97.3%) and MIX (96.9%), and the lowest for the control silage (92.9%). All additives significantly improved the aerobic stability of corn silage, and the greatest value of >72 h was recorded for hetLAB and MIX inoculats, and the lowest for CSC (25 h). In conclusion, additives application can improve fermentation quality, nutritional value, DM recovery and aerobic stability of whole crop corn silage under hot summer conditions of the tropics. The MIX inoculant showed potential to improve in-silo fermentation, and aerobic stability at the same time, however, further investigation are required, particularly with respect of dose rate.

## 1 Introduction

Feeding good quality silage is a pivotal determinant of the profitability of dairy production (Arriola et al., 2021). Whole plant corn is ideal for good quality silage production, due to its high biomass and grain yields, good ensiling characteristics, high metabolizable energy content, and easy incorporation in total mixed ration (Jiang et al., 2022). Moreover, a meta-analysis has shown that incorporation of corn silage in grass or grass silage based diets significantly increases dry matter (DM) intake, and yields of milk and milk protein in dairy cows (Khan et al., 2015). Due to these advantages, new high yielding corn genotypes have been developed for various climatic conditions (Abeysekara et al., 2018), and also for different seasons (spring and summer/autumn) of the year (Jiang et al., 2022). In the changing climatic conditions, selection of suitable corn genotype for quality silage production is one of the most important factors (Khan et al., 2015; Abeysekara et al., 2018), that markedly influence the biomass and nutrient yields, starch: neutral detergent fiber (NDF) ratio, ruminal NDF degradability, site (rumen vs small intestine) of starch digestion, and starch fermentation rate in the rumen (Bal et al., 2000; Khan et al., 2011; Khan et al., 2015).

Although corn silage has good ensiling characteristics, the hot environmental conditions during tropical summer, too high or too low crop DM content at harvest, limitations in the post-harvest processing-technologies and compaction, can significantly deteriorate corn silage fermentation quality and nutritional value (Jiang et al., 2022). Moreover, prolong in-silo fermentation and aerobic exposure during ensiling or feed-out period can increase DM and nutrient losses. Therefore, various additives are used to control the in-silo fermentation process, by compensating limitations of the process through stimulation of desirable fermentation process, but also through prevention of undesirable types of fermentation, resulting in lower DM losses and higher aerobic stability (Muck et al., 2018). Lactic acid producing bacteria (LAB), fermentable substrates (molasses, glucose) and enzymes are used to stimulate desirable fermentation, while organic acids are used to inhibit fermentation (loss of nutrients) by quickly reducing the pH (Oliveira et al., 2017; Bernardi et al., 2019). Among available additives, LAB represents the most popular additives either of those homofermentative LAB (homLAB) or heterofermentative LAB (hetLAB) strains (Muck et al., 2018).

During ensiling, the natural LAB in plants converts sugars under anaerobic conditions to produce lactic acid and acetic acid, which lowers the pH to preserve silage. Fast initial acidification is the key to control the growth of competing enterobacteria, clostridium, yeasts, and molds, and losses of nutrients. However, when the total population and type of natural LAB is not sufficient for the quick fermentation process, then LAB inoculants are usually used to produce high-quality silage by ensuring rapid fermentation (Avila et al., 2014; Bernardes et al., 2018). The LAB has the ability to suppress the growth of undesirable microorganisms and thus reduces the process of proteolysis, and DM loss during the early fermentation phase (Muck et al., 2018; Guan et al., 2020). Furthermore, bacterial inoculants can minimizes mold and yeast growth and improve aerobic stability of silage (Blajman et al., 2018). However, the effects of these LAB inoculants on silage fermentation quality and aerobic stability is highly dependent on the types and species of bacteria (homLAB vs hetLAB) used during silage fermentation (Oliveira et al., 2017).

The homLAB ferment 6-carbon sugars like glucose and fructose to just lactic acid and rapidly decrease silage pH. Therefore, most silage inoculants contain strains of homLAB (Guan et al., 2020). The preferred strains in homLAB inoculants are *Lactobacillus* spp. (*L. plantarum*, *L. acidophilus*, *L. lactis*, *L. bulgaricus*) that can produce a high quantity of lactic acid in a short fermentation period, and stabilizes the silage with minimal nutrients and DM losses (Ni et al., 2015; Wu et al., 2019). On the other hand, hetLAB ferment sugars into both lactic acids and acetic acids. Inoculants containing hetLAB alone typically improve the aerobic stability of silages by fermenting water-soluble carbohydrates (WSC) into organic acids (acetic and propionic acids) that inhibit the growth of aerobic fungi which can cause spoilage to silage, but often slightly increase DM losses and silage pH (Oliveira et al., 2017). The primary and preferred strains in hetLAB inoculants are *L. buchneri* and, less commonly *L. brevis* (Muck et al., 2018).

The primary factor for quality silage production is the composition and abundance of microbial communities during ensiling (Kung et al., 2018; Zi et al., 2021). However, most silage LAB can grow at optimum temperatures of 25 to 40 °C. Ensiling at high temperatures reduces LAB population, lactic acid concentration, fermentation quality and aerobic stability of silage (Guan et al., 2020). Various homLAB and hetLAB additives have been reported to improve silage preservation (Muck et al., 2018; Rabelo et al., 2019), however, their efficiency have not been tested for corn silage production during tropical summer conditions. Moreover, during the corn growth and grain ripening stages, the hot temperature can reduce sugar deposition in corn plants by slowing the rate of photosynthesis through the inactivation of ribulose 1, 5-bisphosphate carboxylase/oxygenase (Bernardes et al., 2018). Therefore, supplementation of molasses alone or in combination with LAB is expected to enhance the fermentation quality of corn grown in the hot climatic conditions.

To our knowledge, the effect of silage additives on corn silage fermentation and nutritional quality under hot summer conditions of the tropics has not been investigated. The recent rise in global temperature further provides impetus to test silage additives for their effectiveness at high ambient temperatures. The first objective of this study was to systematically investigate the effect of additives (homLAB, hetLAB, molasses and their mixture), corn genotypes (Dk6789 and P30K08) and ensiling duration (0, 3, 7, 21, and 150 days) on (1) *in-silo* fermentation characteristics and DM losses; and (2) nutrient composition, Cornell Net Carbohydrate and Protein System (CNCPS) carbohydrate subfractions and *in vitro* digestibility of whole crop corn silage during hot summer (30 to 45°C) conditions. The CNCPS is one the most widely used feed protein and carbohydrate evaluation systems for ruminants (Refat et al., 2017; Xin et al., 2020). The second objective was to evaluated microbial count, and post-ensiling aerobic stability and changes in microbial composition after 150 days ensiling.

## 2 Materials and methods

### 2.1 Corn crop production

**Figure 1:**
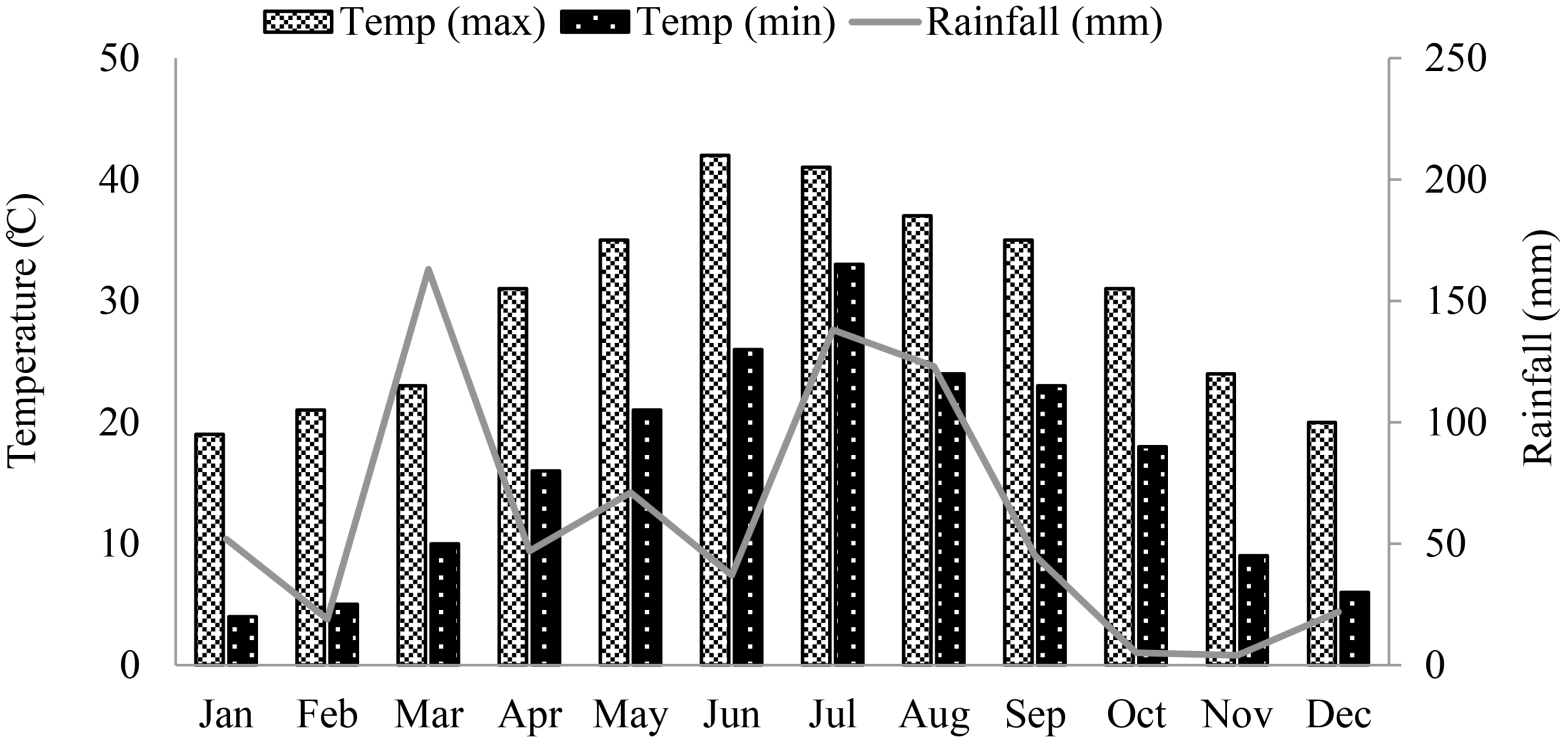
Total monthly rainfall (mm), and minimum and maximum temperature (°C) of the experimental area from January to December 2022. .

Corn was grown during summer season in irrigated research fields of the University of Agriculture Peshawar (34°02′ North Latitude, 71°48′ East longitude, and 347 m above the sea level), Pakistan. The climatological data for the area is shown in **Figure 1**. Seeds of two promising summer corn hybrids, Dk6789 from Monsanto (Monsanto Co. Pvt. Pakistan) and P30K08 from pioneer (Pioneer Hi-Bred International Inc., Pakistan), were sown on June 19, 2022 in four replicate fields. The seeds were sown in ridges (row-to-row spacing of 75 cm, and plant to plant space of 20 cm), at a seed rate of 66,000/ha. Standard, uniform fertilization, irrigation, and weeds control practices were followed for all experimental fields. Based on soil nutrient profile (tested before the experiment), the fields were fertilized with 250, 90 and 90 kg/ha of N, P and K, respectively using di-ammonium phosphate, urea, and sulfate of potash, respectively. The K, P and half of the N fertilizers were applied at the time of sowing and the remaining half of the N fertilizer was applied after first irrigation. For complete weeds control, Prime extra Gold 720SC herbicide was used at a rate of 1200 mL/ha after the first irrigation in a wet field. Besides herbicides, manual weed removal was also carried out, when required. Corn is sensitive to water-stress, and requires frequent irrigation for successful vegetative and reproductive growth under the semi-arid tropical condition. Therefore, all the plots were first irrigated on June 25, 2022, and then irrigated after every two growing weeks. Plant growth was monitored weekly by counting the number of leaves on plants from 1 m randomly selected strip of two consecutive rows of each field, excluding the exterior 1 m area. Similarly, the flowering and silking data was also recorded for better estimation of the harvest date.

### 2.2 Crop harvest, application of inoculant and laboratory-scale silage preparation

The crop was harvested at targeted whole-plant DM content of 33.1%, chopped (with theoretical lengths of cuts ranging from 1.3 to 1.5 cm) with self-propelled forage harvester. The chopped crop of each field was mixed, and a representative samples (100 kg each) were taken. Each sample was treated with the respective inoculant (homLAB, hetLAB, molasses or MIX) or deionized water in case of control corn silage (CCS), and then ensiled in 3 L laboratory silos. Each of the inoculants/deionized water was sprayed on thin layers of chopped corn crop and properly mixed. The homLAB was applied @ 2 mg/kg of fresh forage to supply 1×10^6^ cfu/g of *L. plantarum*, hetLAB inoculant was applied @ of 1 mg/kg fresh forage to supply 4×10^6^ cfu/g of *L. buchneri*, and molasses was added at a 3% of fresh forage. The homLAB inoculant (@ of 2 mg/L) and hetLAB inoculant (@ of 1 mg/L) was dissolved in deionized water and sprayed in a fine mist on the chopped corn. The same amount of deionized water was sprayed over control, without inoculum. The molasses was first diluted in deionized water to easily spray it in a fine mist on the chopped forage. While the MIX treatment contained half of the individual doses of homLAB, hetLAB and molasses, applied with the respective methods as explained above. For each genotype, four subsamples of the control and each of the four inoculated silages were collected and processed as day-0 samples. For each genotype, the CCS, homLAB, hetLAB, molasses, or MIX treated chopped material were ensiled in 16 replicate silos at the density of 260 kg of DM/m^3^. For each corn hybrid, four replicate silos of each treated/control silages were used for each of 3, 7, 21 and 150 days ensiling periods.

### 2.3 Chemical composition

After the specific ensiling durations, the silos containing silage were weighed, opened, and samples were collected for fermentation parameters evaluation, aerobic stability, chemical analysis, carbohydrates subtraction and digestibility. For chemical analysis, subsamples (200 g) of silage from each silo were dried at 70°C in air-dry oven till constant weight. The dried samples were ground in Wiley mill (Thomas Scientific) using 1 mm mesh screen, and analyzed for the contents of DM (method 934.01; AOAC, 2012), ash (method 942.05; AOAC, 2012), ether extract (EE, method 920.39; AOAC International, 2012) and CP (method 981.10; AOAC, 2012). The acid detergent fiber (ADF, method 973.18; AOAC, 2006) and neutral detergent fiber (NDF, method 2002.04; AOAC, 2012) were analyzed without correction for residual CP using Ankom 200 Fiber Analyzer (ANKOM Technology, Macedon, New York). Starch was analyzed using the method of Hall (2015). The neutral detergent-insoluble CP (NDICP) and acid detergent-insoluble CP (ADICP) contents were determined according to the method of Licitra et al. (1996), using ADF and NDF residues. Non-protein nitrogen (NPN) content was analyzed by precipitating the true protein of feed samples with tungstic acid and calculated as the difference between total CP content and CP content of the residues after filtration (Licitra et al., 1996). Soluble CP (SCP) was analyzed by incubating samples with bicarbonate-phosphate buffer for one h at 39°C and filtering the residues through the Whatman #54 filter paper. The SCP content was calculated as the difference between the total CP content and the CP content in the residues. The non-fiber carbohydrate (NFC) was calculated as NFC = 100 – (NDF – NDICP) – EE – CP – ash (NRC, 2001).

### 2.4 Fermentation quality, microbial composition and aerobic-stability analysis

#### 2.4.1 Fermentation quality

For measurement of fermentation quality after 3, 7, 21, 150 days of ensiling, 50 g of fresh, unground sample form each replicate silo was dissolved in 450 mL of distilled water. The mixture was sealed, blended with high speed blender until complete homogenization. Immediately after blending, the pH was measured by pH meter (AE150 pH Benchtop meter; Thermo Fisher Scientific Inc.). The homogenate was filtered through two layers of cheesecloth. One aliquot of the filtrate was centrifuged at 10,000×g for 10 min at 4°C, and analyzed for ammonia-N (NH_3_-N), and organic acids contents. The NH_3_-N was analyzed by colorimetry using an auto-analyzer (Technicon; now SEAL Analytical, Hampshire, UK). The contents of organic acids, including lactic acid, acetic acid, propionic acid and butyric acid, was analyzed using high-performance liquid chromatography (Hitachi, L-2400, Ibaraki, Japan), equipped with a UV detector (wavelength 210 nm; Hitachi L-2400), autosampler (Hitachi Autosampler L-2200), and an Aminex HPX87H column (Bio-Rad Laboratories) with 0.015 M sulfuric acid mobile phase, run at a flow rate of 0.7 mL/min at 47°C.

#### 2.4.2 Enumeration of microorganisms

The second aliquot of filtrate of each sample was immediately used for measuring total LAB, yeast, and mold counts by using the pour plate method. The yeast and mold were enumerated after 72 h aerobic incubation at 32°C in malt extract agar (Thermo Fisher Scientific-Oxoid CM0059B). The malt extract agar was acidified (after autoclaving) with 85% lactic acid at rate of 0.5% (vol/vol) of liquid agar medium, to inhibit growth of bacteria. The numbers of viable yeast and mold colonies were counted. The numbers of LAB were determined using the pour plate method with de Man Rogosa Sharpe agar (Thermo Fisher Scientific R01585), and incubated anaerobically at 32°C for 48 h.

### 2.5 DM recovery and aerobic-stability analysis

The DM recovery after 150 days of ensiling was calculated using the DM content and the weight of the forage placed in the silo before ensiling, and on the day of the opening as follows:


$$DM\;recovery=\frac{\left(FCX\;\times DMFX\right)}{\left(SSX\times DMSX\right)}$$

Where FCX is weight of fresh whole crop corn forage placed in the silo X, DMFX is the DM content of the fresh forage placed in the silo; SSX is the weight of silage in the silo X after 150 days, and MDSX is the DM content of the silage in silo X.

In the current study the silages were exposed to air at a high ambient temperature (36 ± 1°C) for determination of aerobic stability after, 150 days ensiling. Aerobic stability was defined as the time (hours) required for increasing the temperature of aerobically exposed silage by 2°C above the ambient temperature. Two kg silage from each replicate silo were placed in 8 L buckets, mixed for complete aeration, and kept in a room with a controlled environment. The temperature was measured every 15 min using two temperature sensors, placed in the geometric center of the silage. Buckets were covered with two layers of sterile cheesecloth to avoid contamination and drying out of the silage, yet allowing infiltration of air to the silage. Three additional sensors were placed in the room to record ambient temperature. The temperature was collected using a multipoint real-time temperature recorder (Mike Sensor Technology Co., LTD., Hangzhou, Zhejiang, China). The numbers of lactic acid bacteria, yeast, aerobic bacteria and mold were also measured as spoilage parameters after aerobic exposure.

### 2.6 Carbohydrates sub fraction and *in vitro* digestibility

The carbohydrates subtractions were computed using the updated version of the Cornell Net Carbohydrate and Protein System (CNCPS; Higgs et al., 2015; Van Amburgh et al., 2015). The carbohydrates were fractionated into CA1-subfraction (volatile fatty acids), CA2-subfraction (lactic acids), CA3-subfraction (organic acids), CA4-subfraction (soluble sugars), CB1-subfraction (starch), CB2-subfraction (soluble fiber), CB3-subfraction (available NDF), and CC-subfraction (unavailable NDF). The degradation rates (Kd) of the different subfractions in the rumen are: 0/h for CA1, 0.7/h for CA2, 0.5/h for CA3, 0.40–0.60/h for CA4, 0.20–0.40/h for CB1, 0.20–0.40/h for CB2, 0.04–0.09/h for CB3, and CC is non-degradable subfraction.

The two-stage *in vitro* procedure was adopted for determination of the *in vitro* DM digestibility (DMD) as reported earlier by Khan et al. (2022).

### 2.7 Statistical analysis

The effects of inoculum, genotypes, and ensiling duration on silage fermentation characteristics, chemical composition, carbohydrate subfractions, and DMD were determined by repeated measure analysis of variance using the PROC MIXED procedure of SAS (SAS Inst., Inc., Cary, NC). The different covariance structures of repeated matrices were evaluated according to Littell et al. (2006) and Wang and Goonewardene (2004) using the Akaike information criterion and the Schwarz Bayesian criterion. The following model was used:

Yijkl = μ + Ii + CVj + EDk + Ii × EDj+ ϵijkl

Whereas, Yijkl is the response of the treatment, µ is the overall mean, Ii is the fixed effect of inoculum, CVj is the fixed effect of cultivars of silage corn, EDk is the fixed effect of ensiling duration, Ii × EDj is the effect of interaction of inoculums and ensiling duration (only significant and interesting interactions are presented in the results), and ϵijkl is the random error. The effects of inoculum type on DM recovery, aerobic stability and number of microorganisms was determined using Proc Mixed procedure of SAS. Genotype and replication were considered as random effects. *Post-hoc* analyses were carried out using the Tukey−Kramer test to compute pair wise differences in the means. Means with different letters were obtained with “pd mix 800SAS macro”.

## 3 Results

### 3.1 Chemical profile and digestibility (*in vitro*) of corn silage

Data on the overall effects of silage additives, ensiling duration and corn genotypes on the proximate chemical composition, CP and carbohydrates chemical profiles and DMD (*in vitro*) of whole crop corn silages are summarized in **Table 1**. Except lignin and starch, the mean contents of all measured chemical components of whole crop corn silage were significantly (*P* < 0.01) altered by the additives. Compared to CCS, all additives treated silages had higher (*P* < 0.05) contents of CP, NDICP, ADICP, NFC and DMD, and lower contents of SCP, ADF and NDF. Notably, among the inoculated groups the variation in contents on CP (6.94-7.13% DM), SCP (24.0-26.3% CP), NDICP (22.2-23.6% CP), ADICP (6.63-7.36% CP), NFC (48.3-49.8% DM) and DMD (63.9-66.4%) were quantitatively small. The homLAB and MIX inoculated silages had the highest (*P* < 0.05) contents of DM, CP, NFC and DMD, and lowest (*P* < 0.05) content of SCP.

**Table 1:** Effect of silage additives, ensiling duration and genotypes on chemical profile, protein and carbohydrates chemical profiles and digestibility (*in vitro*) of whole crop corn silages.

	DM (%FM)	CP (%DM)	SCP (%CP)	NDICP (%CP)	ADICP (%CP)	WSC (%DM)	NFC (%DM)	Lignin (%DM)	ADF (%DM)	NDF (%DM)	Starch (%DM)	DMD (%)
Silage additives												
Control	33.1^c^	6.66^c^	27.9^a^	21.2^d^	5.64^d^	2.42^c^	47.4^c^	2.93	23.3^a^	42.6^a^	30.0	63.1^d^
HomLAB	34.6^a^	7.13^a^	24.0^c^	23.6^a^	7.36^a^	3.53^a^	49.8^a^	2.88	21.7^c^	40.9^c^	30.9	66.4^a^
HetLAB	33.8^b^	7.00^b^	26.3^b^	22.6^bc^	6.96^b^	2.63^c^	48.3^b^	3.06	22.5^b^	41.8^b^	30.6	63.9^c^
Molasses	33.7^b^	6.94^b^	25.8^b^	22.2^c^	6.63^c^	2.90^b^	48.7^b^	2.95	22.8^b^	41.7^b^	31.5	65.2^b^
Mixture	34.2^ab^	7.06^ab^	25.9^b^	22.9^b^	6.94^b^	3.29^ab^	49.4^ab^	3.01	22.9^ab^	42.3^ab^	31.0	65.8^ab^
SEM	0.18	0.06	0.31	0.21	0.161	0.21	0.39	0.02	0.24	0.38	0.24	0.33
Ensiling duration (days)												
D0	35.0^a^	7.33^a^	12.6^e^	20.6^a^	7.19^a^	6.47^a^	49.8^a^	2.99	22.7	43.7	32.1	60.9^d^
D7	34.1^b^	7.14^b^	21.5^d^	19.6^b^	7.18^a^	2.71^b^	49.3^b^	2.98	22.7	43.6	32.0	62.9^c^
D21	33.5^c^	6.95^cd^	26.3^c^	19.6^b^	6.89^ab^	2.52^b^	48.7^c^	2.96	22.7	43.7	32.2^c^	64.7^b^
D42	33.2^cd^	6.68^d^	31.4^b^	18.9^bc^	6.58^b^	2.19^c^	48.2^d^	2.94	22.6	43.8	32.2	65.7^a^
D150	32.9^d^	6.66^d^	36.9^a^	18.7^c^	6.30^b^	2.05^c^	47.4^e^	2.91	22.6	43.9	32.3	65.8^a^
SEM	0.17	0.10	0.360	0.19	0.150	0.19	0.24	0.02	0.254	0.48	0.28	0.13
Corn genotypes												
P30K08	33.4^b^	6.75^b^	24.9^b^	19.3	6.94	3.01	51.0^a^	2.89^b^	22.1	39.5^b^	33.9^a^	64.3^a^
Dk6789	34.1^a^	7.09^a^	26.6^a^	19.7	6.72	3.02	45.9^b^	3.03^a^	23.1	42.9^a^	28.3^b^	63.2^b^
SEM	0.05	0.015	0.05	0.13	0.10	0.11	0.25	0.01	0.15	0.24	0.14	0.04
Significance												
Inoculums	***	***	***	***	***	***	**	NS	**	**	NS	***
ED	***	***	***	***	***	***	***	NS	NS	NS	NS	***
Genotype	***	***	***	NS	NS	NS	***	***	NS	***	***	*

Mean with different superscription (^a-e^) in the same column within silage inoculants, ensiling duration or corn genotypes differ at P < 0.05.

homLAB, inoculated with homofermentative lactic acid producing bacteria (LAB) (2 mg/kg fresh forage to supply 1×10^6^ cfu/g of Lactobacillus plantarum); hetLAB, inoculated with heterofermentative LAB (1 mg/kg of fresh forage to supply 4×10^6^ cfu/g of L. buchneri); Mol, inoculated with molasses (3% of fresh forage); MIX, inoculated with combination of half of the individual doses of hetLAB, hetLAB and molasses inoculants; ED, ensiling days; DM, dry matter; CP, crude protein; SCP, Soluble CP; NDICP, neutral detergent insoluble CP; ADICP, Acid detergent insoluble CP; WSC, water soluble carbohydrates; NFC, non-fibrous carbohydrates; ADF, acid detergent fiber; NDF, neutral detergent fiber; DMD, dry matter digestibility; SEM, standard error of mean; NS, non-significant; ***P < 0.001; **P < 0.01; *P < 0.01.

The mean contents of all measured chemical components altered (*P* < 0.001) with the length of ensiling period (0-150 days), except ADF, NDF, lignin and starch (**Table 1**). The contents of DM (35.0 to 32.9%), CP (7.33 to 6.66% DM), NDICP (20.6 to 18.7% CP), ADICP (7.19 to 6.30% CP) and NFC (49.8 to 47.4% DM) consistently decreased (*P* < 0.05) with the increase in ensiling duration from 0 to 150 days. In contrast, the content of SCP (12.6 to 36.9% CP) and DMD (60.9 to 65.8%) increased (*P* < 0.05) during the 150 days ensiling. Among the corn genotypes, silages produced from P30K08 had greater (*P* < 0.05) content of NFC (51.0 vs 45.9% DM), starch (33.9 vs 28.3% DM), and *in vitro* DMD (64.3 vs 63.2%) as compared to DK6789.

### 3.2 CNCPS carbohydrates subfractions

Data on the overall effect of silage additives, ensiling duration, and corn genotypes on the CNCPS carbohydrates subfraction composition of the corn silage are presented in **Table 2**. All the reported CNCPS subfractions significantly varied (*P* < 0.001) due to the application of additives, except CB1, CB3 and CC subfractions. Compared to CCS, all additives increased (*P* < 0.05) of CA1, CA2 and CA4 subfractions, and decreased (*P* < 0.05) CB2 subfraction. Among the additives, the homLAB produced corn silage with the highest (*P* < 0.05) content of CA2 (9.20% DM; Kd 0.7/h) and CA4 (2.82% DM; Kd 0.40–0.60) subfractions, and lowest contents of CA1 (1.70% DM; Kd 0/h) and CB2 (2.73% DM; Kd 0.20–0.40/h) subfractions, demonstrating desirable and restricted in-silo fermentation. On the other hand, the hetLAB inoculated silages had the greatest (*P* < 0.05) value of CA1-subfraction, highlighting greater production of organic acids, required for aerobic stability. Interestingly, the MIX silages followed the hetLAB in CA1 value and homLAB in CA2 value. With the increase in ensiling duration from 3 to 150 days, there were consistent decline in CA1 (2.50 to 1.74% DM), CA4 (2.52 to 2.02% DM) and CB2 (39.5 to 37.9% DM) CNCPS subfractions. In contrast the CA2-subfraction increased from 5.42 to 8.82% DM during the 150 days ensiling period. Among the corn genotypes, P30K08 had greater (*P* < 0.05) CB1 (33.8 vs 28.3% DM), and lower CB3 (36.4 vs 41.3) and CC (2.71 vs 3.23% DM) subfractions as compared to DK6789.

**Table 2:** Effect of silage additives and ensiling duration on Cornell Net Carbohydrate and Protein System (CNCPS) subfractions of whole crop corn silage.

	CA1	CA2	CA4	CB1	CB2	CB3	CC
Silage Inoculants							
Control	1.41^e^	5.92^e^	2.01^d^	30.0	8.86^a^	39.5	3.02
homLAB	1.70^d^	9.20^a^	2.82^a^	30.9	2.73^d^	39.5	2.92
hetLAB	2.92^a^	6.21^d^	2.28^c^	30.6	7.89^b^	38.7	3.04
Molasses	2.03^c^	6.96^c^	2.57^b^	31.5	6.01^c^	38.3	2.93
Mixture	2.45^b^	7.35^b^	2.33^c^	31.0	6.71^bc^	38.7	2.96
SEM	0.09	0.055	0.030	0.24	0.456	0.39	0.04
Ensiling duration							
ED3	2.50^a^	5.42^d^	2.92^a^	32.1	7.12^a^	39.5^a^	3.04
ED7	2.10^b^	6.74^c^	2.88^a^	32.0	6.87^a^	39.3^a^	3.03
ED21	2.08^b^	7.55^b^	2.19^b^	32.2	6.36^ab^	38.5^b^	2.96
ED150	1.74^c^	8.82^a^	2.02^c^	32.2	5.59^b^	37.9^c^	2.86
SEM	0.15	0.55	0.028	0.214	0.40	0.35	0.092
Genotype							
P30K08	2.13	7.10	2.40	33.8^a^	6.28	36.4^b^	2.71^a^
DK6789	2.10	7.15	2.39	28.3^b^	6.60	41.3^a^	3.23^b^
SEM	0.12	0.35	0.08	0.14	0.288	0.248	0.024
Significance							
Inoculums	***	***	***	NS	***	NS	NS
Ensiling duration	***	***	***	NS	*	**	NS
Genotype	NS	NS	NS	***	NS	***	***

Mean with different superscription (^a-e^) within column within silage inoculants, ensiling duration and corn genotypes differ at P < 0.05. homLAB, inoculated with homofermentative lactic acid producing bacteria (LAB; 2 mg/kg fresh forage to supply 1×10^6^ cfu/g of Lactobacillus plantarum); hetLAB, inoculated with heterofermentative LAB (1 mg/kg of fresh forage to supply 4×10^6^ cfu/g of L. buchneri); Mol, inoculated with molasses (3% of fresh forage); Mix, inoculated with MIX (combination of half of the individual doses of hetLAB, hetLAB and molasses) inoculants. CA1, wolatile fatty acids (Kd 0/h); CA2, lactic acid (Kd 0.7/h); CA4, sugar (Kd 0.40–0.60/h); CB1, starch (Kd 0.20–0.40/h); CB2, soluble fiber (Kd 0.20–0.40/h); CB3, digestible fiber (Kd 0.04–0.09/h); CC, indigestible fiber; ED, ensiling days; SEM, standard error of mean; NS, non-significant; *p < 0.05; **p < 0.01; ***p < 0.001.

### 3.3 Fermentation quality of whole crop corn silage

**Figure 2:**
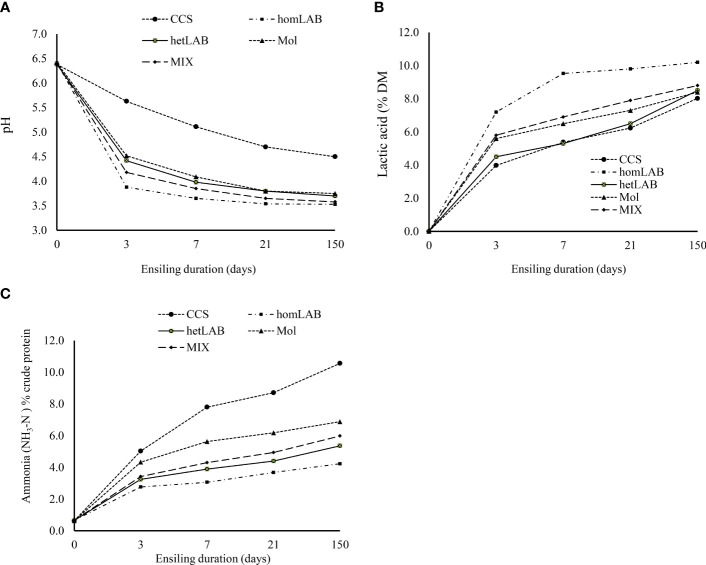
Changes in pH **(A)** ammonia [NH_3_-N; **(B)**] and lactic acids **(C)**] contents during 150 days ensiling of whole crop corn, as affected by the different inoculants. CCS, control corn silage; homLAB, inoculated with homofermentative lactic acid producing bacteria (LAB; 2 mg/kg fresh forage to supply 1×10^6^ cfu/g of *Lactobacillus plantarum)*; hetLAB, inoculated with heterofermentative LAB (1 mg/kg of fresh forage to supply 4×10^6^ cfu/g of *L. buchneri*); Mol, inoculated with molasses (3% of fresh forage); MIX, inoculated with a mixture of half of the individual doses of homLAB, hetLAB and molasses inoculants. .

Data in **Table 3** and **Figure 2** illustrate the fermentation quality whole crop corn silage treated with different additives and ensiled for different durations. The applications of different additives altered the contents of lactic acids (*P* < 0.001), acetic acids (*P* < 0.001), NH_3_-N (*P* < 0.001) and pH (*P* < 0.01) of corn silage. Compared to CCS, application of additives increased (*P* < 0.05) the contents of lactic acids and acetic acids and decreased (*P* < 0.05) the content of NH_3_-N and pH. Among the additives, homLAB inoculated silage had the highest (*P* < 0.05) content of lactic acid (9.20% DM), and lowest (*P* < 0.05) content of acetic acid (1.16% DM), NH_3_-N (3.46% N) and pH (3.66). The highest (*P* < 0.05) value of acetic acid was observed for hetLAB (2.01% DM), followed by MIX (1.41% DM). With the increase in ensiling duration from 3 to 150 days, the lactic acids (5.42 to 8.82% DM) and NH_3_-N (5.42 to 8.82% DM) increased (*P* < 0.05), whereas, acetic acid (1.88 to 1.05% DM) and pH (4.54 to 3.69) decreased (*P* < 0.05). Corn genotypes, did not alter any of the measured silage fermentation characteristics.

**Table 3:** Effect of silage additives and ensiling duration on fermentation characteristics of whole crop corn silage.

	Lactic acid (% DM)	Acetic acid (% DM)	Propionic Acid (% DM)	NH_3_-N (%N)	pH
Silage inoculants					
Control	5.92^e^	0.91^e^	0.10	8.08^a^	4.99^a^
^hom^LAB	9.20^a^	1.16^c^	0.00	3.46^e^	3.66^c^
^het^LAB	6.21^d^	2.01^a^	0.10	4.22^c^	3.94^b^
Molasses	6.96^c^	1.31^bc^	0.15	6.27^b^	3.91^b^
Mix	7.35^b^	1.41^b^	0.20	3.89^d^	3.76^b^
SEM	0.055	0.09	0.12	0.20	0.133
Ensiling duration (days)					
ED3	5.42^d^	1.88^a^	ND	3.76^d^	4.54^a^
ED7	6.74^c^	1.50^b^	0.10	4.96^c^	4.12^b^
ED21	7.55^b^	1.00^c^	0.20	5.70^b^	3.84^bc^
ED150	8.82^a^	1.05^c^	0.35	6.95^a^	3.69^c^
SEM	0.055	0.10	0.10	0.12	0.13
Corn genotypes					
P30K08	7.15	1.36	0.20	5.36	4.07
Dk6789	7.10	1.35	0.10	5.31	4.04
SEM	0.35	0.05	0.10	0.06	0.02
Significance					
Inoculums	***	***	NS	***	**
Ensiling time	***	***	NS	***	***
Genotype	NS	NS	NS	NS	NS

Mean with different superscription (^a-e^) in the same column within silage inoculants, ensiling duration or corn genotypes differ at P < 0.05. homLAB, inoculated with homofermentative lactic acid producing bacteria (LAB; 2 mg/kg fresh forage to supply 1×10^6^ cfu/g of Lactobacillus plantarum); hetLAB, inoculated with heterofermentative LAB (1 mg/kg of fresh forage to supply 4×10^6^ cfu/g of L. buchneri); Mol, inoculated with molasses (3% of fresh forage); Mix, inoculated with MIX (combination of half of the individual doses of hetLAB, hetLAB and molasses) inoculants. NH_3_-N; ammonia nitrogen; ED, ensiling days; ND, not determined; DM, dry matter; SEM, standard error of mean; NS, non-significant; **P < 0.01; ***P < 0.001.

Changes in pH (Panel A), NH_3_-N (Panel B) and lactic acids (Panel C) contents during 150 days ensiling of whole crop corn, as affected by the different inoculants are depicted in **Figure 2**. It is evident from **Figure 2** that without inoculum stable pH was not achieved even 21 days after ensiling. However, with application of molasses, hetLAB and the MIX inoculants, stable pH was achieved during 7 days of ensiling, and with homLAB stable pH was achieved during the first 3 days of ensiling. The rapid drop in pH with homLAB was associated with the greater increase in lactic acid concentration (0 to 7.20% DM) during the first 3 days of ensiling (**Figure 2B**). At all ensiling durations, the highest values of lactic acids were recorded for homLAB, followed by MIX (**Figure 2B**). Moreover, the lowest increase in NH_3_-N concentration (0.66 to 4.23% CP) during the 150 days ensiling was recorded for homLAB treated corn silage (**Figure 2C**). In contrast, the CCS had the greatest values of NH_3_-N concentration at all ensiling durations, followed by molasses, MIX and hetLAB inoculated silages.

### 3.4 Microbial population, dry matter recovery and aerobic stability of whole crop corn silage

**Figure 3:**
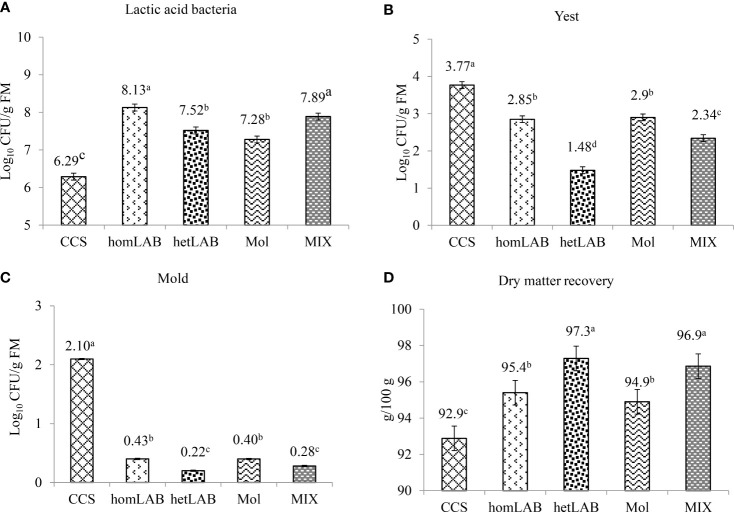
Effects of different inoculants on population of lactic acid bacteria **(A)**, yeast **(B)** mold **(C)** and dry matter recovery (Panel **D**) of whole crop corn ensiled for 150 days. CCS, control corn silage; homLAB, inoculated with homofermentative lactic acid producing bacteria (LAB; 2 mg/kg fresh forage to supply 1×10^6^ cfu/g of *Lactobacillus plantarum)*; hetLAB, inoculated with heterofermentative LAB (1 mg/kg of fresh forage to supply 4×10^6^ cfu/g of *L. buchneri*); Mol, inoculated with molasses (3% of fresh forage); MIX, inoculated with a mixture of half of the individual doses of homLAB, hetLAB and molasses inoculants.

**Figure 3** show the effects of different inoculants on the total number of viable LAB (Panel A), yeasts (Panel B), mold (Panel C) and DM recovery (Panel D) of whole crop corn ensiled for 150 days. All the tested inoculants significantly improved (*P* < 0.001) the count of LAB and DM recovery, and decreased (*P* < 0.001) the count of yeast and mold. Notably, the greatest number of viable LAB was (*P* < 0.05) recorded in homLAB (8.13 log cfu/g) and MIX (7.89 log cfu/g) silages, while the lowest (*P* < 0.05) number was recorded for CCS (6.29 log cfu/g). The lowest (*P* < 0.05) yeast counts were recorded in hetLAB (1.48 log cfu/g) inoculated silage, and the highest (*P* < 0.05; 3.77 log cfu/g) for the CCS (**Figure 3B**). The highest (*P* < 0.05) count of mold was recorded for the CCS (2.10 log cfu/g), and the lowest (*P* < 0.05) for hetLAB (0.22 log cfu/g) and MIX (0.28 log cfu/g). The DM recovery of whole crop corn silage was significantly improved (*P* < 0.001) with the application of the different inoculants (**Figure 3D**). The greatest (*P* < 0.05) DM recovery (97.3%) was recorded for hetLAB and MIX (96.9%), and the lowest (*P* < 0.05) for the control silage (92.9%).

**Figure 4:**
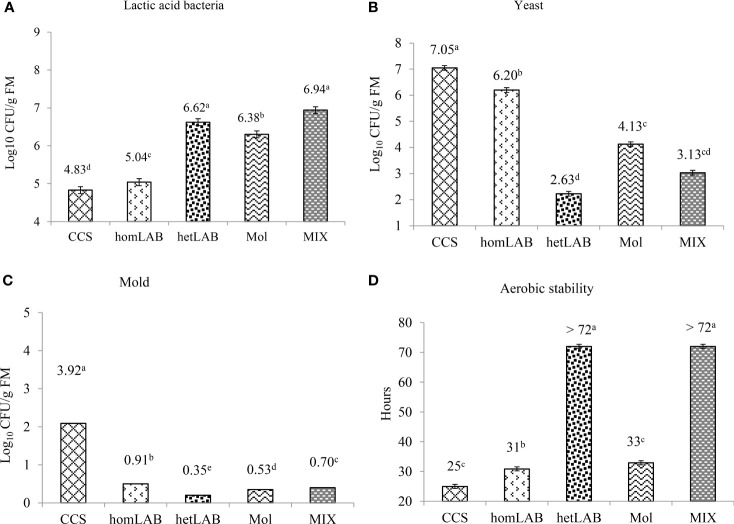
Effects of different inoculants on population of lactic acid bacteria **(A)** yeast **(B)** mold **(C)**) and aerobic stability **(D)** after 72 h exposure to air of the 150 days ensiled whole crop corn. CCS, control corn silage; homLAB, inoculated with homofermentative lactic acid producing bacteria (LAB; 2 mg/kg fresh forage to supply 1×10^6^ cfu/g of *Lactobacillus plantarum)*; hetLAB, inoculated with heterofermentative LAB (1 mg/kg of fresh forage to supply 4×10^6^ cfu/g of *L. buchneri*); Mol, inoculated with molasses (3% of fresh forage); MIX, inoculated with a mixture of half of the individual doses of homLAB, hetLAB and molasses inoculants.

**Figure 4** shows the number of viable LAB (Panel A), yeasts (Panel B), mold (Panel C) and aerobic stability (Panel D) after 72 h exposure to air of the 150 days ensiled whole crop corn. All the tested inoculants significantly improved (*P* < 0.001) the count of LAB and aerobic stability, and decreased (*P* < 0.001) the count of yeast and mold. The highest (*P* < 0.05) numbers of viable LAB were counted for homLAB (7.04 log cfu/g) and MIX (6.94 log cfu/g) inoculated silages after the 72 h exposure to air, and the lowest (*P* < 0.05) for CCS (4.83 log cfu/g). The lowest (*P* < 0.05) number of viable yeasts (2.63 log cfu/g) and mold (0.35 log cfu/g) were counted for hetLAB inoculated silages. The highest aerobic stability of >72 h was recorded for hetLAB and MIX inoculated corn silages. Silages inoculated with homLAB were stable for 31 h and those with molasses were only stable for 33 h. Nevertheless, all the inoculated corn silages had greater aerobic stability than the CSC (25 h).

## 4 Discussion

High ambient temperature during the grain filling maturity (Bernardes et al., 2018), ensiling (Ali et al., 2015) and feed-out period (Khan et al., 2009; Ferrero et al., 2021) strongly influence the chemical composition, epiphytic microbial population, *in-silo* fermentation characteristics, and aerobic stability of corn silage (Guan et al., 2020; Ferrero et al., 2021). Therefore, global warming has been envisaged as a major challenge for future silage production, particularly in the tropics (Guan et al., 2020). The results of this study present the first dataset on the effects of homLAB (*L. plantarum)*, hetLAB (*L. buchneri*), molasses and their mixture on the chemical composition, ensiling quality, DM losses, CNCPS carbohydrate subfractions, *in vitro* digestibility, microbial count, and post-ensiling aerobic stability of corn silage during hot summer conditions of the tropics.

During *in-silo* fermentation, sugars and other easily fermentable carbohydrates are converted to volatile fatty acids, lactic acid, and CO_2_ by microbes and plant enzymes (Oliveira et al., 2017; Muck et al., 2018; Bernardi et al., 2019). The process results in DM and energy loss, which reduces the availability of fermentable carbohydrates for ruminal fermentation (Cone et al., 2008). As such, faster LAB fermentation, and establishment of low (< 4.2; McDonald et al., 1991) pH is important for stabilization of the silage, and for avoiding undesirable microbial growth and losses due to prolong and undesirable fermentation (Muck et al., 2018). For a quicker and quality fermentation the content of WSC and epiphytic LAB are very important. However, high ambient temperature can reduce the WSC (Bernardes et al., 2018) and population of epiphytic LAB in corn silage (Muck et al., 2018; Guan et al., 2020), and increase the production of acetic acid, which slow-down pH decline and increase DM and energy loss (Bernardes et al., 2018). Therefore, in current study LAB inoculants, molasses and their mixture were used to stimulate a rapid decline in pH, inhibit the growth undesirable anaerobic microorganisms, and prevent prolonged fermentation, extensive proteolysis and DM loss during the ensiling process (Ferrero et al., 2018; Arriola et al., 2021).

In the current study, DM content of the whole crop corn silages ranged from 33.1 to 34.6%, which is within the optimal range (Khan et al., 2015). All additives improved nutrient profile and digestibility of corn silage. The highest improvement in DM, CP, NDICP, WSC and NFC contents were recorded for homLAB inoculated silages. The homLAB stimulated fast homo-lactic acid fermentation, resulting in a rapid decline in pH and lower losses of fermentable carbohydrates. Moreover, the fast establishment (within 3 days) of lower pH (3.88) prevented the growth undesirable anaerobic microorganism and extensive proteolysis in the homLAB inoculated silage, which can explain the high DM content, and improvement in nutrient profile (Oliveira et al., 2017; Muck et al., 2018). In agreement with our findings earlier studies have reported greater DM content, and better nutritional value for corn silages inoculated with homLAB (Bernardi et al., 2019; Zhang et al., 2022). The lower content of CP, NDICP and WSC, and higher content of SCP in the CCS reflects prolonged/undesirable fermentation and extensive proteolysis (Oliveira et al., 2017). The contents of NDF ranged from 41.7 to 42.6% DM, with the lowest value recorded for homLAB-treated silage. In agreement with our findings earlier studies have reported that the application of homLAB reduces the NDF content (by up to 5%) by decreasing cell wall recalcitrance during the fermentation (McDonald et al., 1991; Jalc et al., 2009). For quality silage fermentation, the presence of WSC content (> 5% DM) is necessary (McDonald et al., 1991; Zi et al., 2021). In the present study the WSC of the unensiled whole crop corn was 6.47% DM, which was enough for the ensiling process, and this could have probably diminished the positive effects of molasses additives on corn silage nutritional quality. The homLAB-treated silages had the highest content of WSC as compared to the other groups, highlighting better/restricted fermentation and lower losses. Our results are in line with Huisden et al. (2009), who reported higher content WSC in silage inoculated with homLAB. All additives improved DMD of corn silage. The DMD of homLAB inoculated corn silage was 4.7% greater than the CCS. Similar increases in DMD were found in earlier studies where homLAB was applied to silages, supporting our findings (Huisden et al., 2009). High digestibility of forages is one of the most desirable characteristics for proper diet formulation and animal performance, as digestibility of forages is closely associated with intake, energy and nutrients supply, and animal performance (Khan et al., 2015). Notably, the nutritional value of the MIX silage was not significantly different from that of homLAB inoculated silage. The MIX silage did not exceed the homLAB in terms of silage nutritional value, which may be related to the lower doses of individual additives used in the MIX inoculant. In agreement with our findings, a recent review reported inconsistent effects of MIX inoculants on silage nutritional value (Muck et al., 2018).

Irrespective of the additives application, ensiling duration had a significant effect on the chemical composition, protein, and carbohydrates chemical profiles. The CP content decreased from 7.09 to 6.66% during the 150 days ensiling period. In agreement with our findings, earlier studies reported that the CP content decreased by 2-3% with advancing ensiling duration (Cone et al., 2008; Silva et al., 2015). A large fraction of WSC was consumed by LAB during the fermentation process, which can explain the decrease in its concentration from 6.47 to 2.05% DM during the 150 days ensiling. During the 150 days ensiling, the NDF content decreased by 3%. Gerlach et al. (2013) and Silva et al. (2015) reported a similar decrease (2-5%) in the NDF content with the increase in ensiling duration from 1 to 60 days. During the ensiling and fermentation processes, enzymatic and acid hydrolysis of the highly digestible cell wall portion, hemicellulose, may account for the decrease in NDF content (Junges et al., 2013). The DMD increased by 8.05% with the increase in ensiling duration from 0 to 150 days. Since the easily fermentable carbohydrates fractions of the silages decreased with the increase in ensiling duration, the increase in digestibility could be related to the increase in protein solubility and the positive effects on ensiling on degradability of the insoluble protein and carbohydrates fractions.

The CNCPS (Higgs et al., 2015; Van Amburgh et al., 2015), is widely used for evaluation of feed protein and carbohydrates nutritional value for ruminants, and for diet formulation according to dairy cattle requirements (Refat et al., 2017; Sun et al., 2018). The CNCPS subfractions (CB1, CB2, CB3 and CC) reported in this study were close to the reported values of Jiang et al. (2022). All inoculated silages had higher CA1 (Kd 0/h), CA2 (Kd 0.7/h) and CA4 (Kd 0.40–0.60) subfractions, demonstrating that the use of additives increased the contents of rapidly fermentable carbohydrates in corn silage. In agreement with our findings, earlier studies have reported greater CA-subfractions for LAB inoculated silages (Higgs et al., 2015). It may be noted that the CNCPS subfractions are calculated from the chemical compositions data (Xin et al., 2020b), and as such, the variations observed on the CNCPS are strongly explained from the differences in chemical composition due to the additives, ensiling duration and genotypes. The highest CA2 (9.20% DM) and CA4 (2.82% DM) subfractions, and lowest contents of CA1 (1.70% DM) and CB2 (2.73% DM; Kd 0.20–0.40/h) subfractions in the homLAB silages, are the consequence of faster fermentation and quicker establishment of low pH. On the other hand, the hetLAB inoculated silages had the greatest (P < 0.05) value of CA1-subfraction, highlighting greater production of organic acids, required for aerobic stability. Interestingly, the MIX silages followed the hetLAB in CA1 value and homLAB in CA2 value. These findings highlight that there is a potential to get the benefits of desirable in-silo fermentation of homLAB, and aerobic stability of hetLAB from the MIX inoculants. However, more work is needed to develop proper mixture.

A lower pH (< 4.2; McDonald et al., 1991) is an important index of good silage preservation (Rabelo et al., 2019). In the current study, without inoculum the pH was not stable (>4.2), even after 21 days of ensiling. However, with the application of molasses (4.09) and hetLAB (3.87) stable pH was achieved during 7 days of ensiling, and with homLAB (3.88) and MIX (4.18) stable pH was achieved during the first 3 days of ensiling. In agreement with our findings, earlier studies have reported lower pH values for homLAB inoculated silages (Oliveira et al., 2017). The rapid drop in pH was associated with the greater increase in lactic acid concentration in homLAB (0 to 7.20% DM) and MIX (0 to 5.8% DM) during the first 3 days of ensiling (**Figure 2B**). The rapid decrease in pH inhibits the growth of (spoilage)-microorganisms, thus reducing the proteolysis and butyric acids production, and preserve nutritional value of silage (McDonald et al., 1991; Ogunade et al., 2016). In currents study inoculation with homLAB increased the lactic acid production and reduced acetic acid and other acids which is consistent with literature findings (Oliveira et al., 2017). However, the lower content of acetic acid and other organic acids with homLAB can reduce yeast count and aerobic stability of silage (Oliveira et al., 2017; Muck et al., 2018). The hetLAB are mainly producing hetero fermentative acids, lactic acid, acetic acid, ethanol and CO_2_ that reduces pH slowly as compared to homLAB (Oliveira et al., 2017). This could explain the highest content of acetic acid in hetLAB inoculated silages in present study. Moreover, the high content of acetic acid is favored for increasing aerobic stability due to its potential to inhibit yeasts, responsible for initiating aerobic spoilage (Muck et al., 2018). Notably, like homLAB, inoculation with MIX inoculant significantly improved the rate and extent of pH decline and lactic acid production. On the other hand, MIX inoculant significantly improved acetic acid production. Although, in the current study the positive effects of MIX did not exceed, the individual (hom/het LAB) inoculants, it shows potential to improve in-silo fermentation and aerobic stability at the same time and needs further investigation, particularly with respect of dose rate.

The concentration of NH_3_-N, reflects the extent of proteolysis, and amino acid deamination and decarboxylation during ensiling (Oliveira et al., 2017). During in silo fermentation, protein hydrolysis can be inhibited by establishment of low pH environment, which prevent the growth of proteolytic microorganisms and ceases the activity of proteolytic enzymes. All additives significantly decreased the concentration of NH_3_-N by accelerating the rate and extent of pH decrease during ensiling. The homLAB inoculated silage had the lowest concentration of NH_3_-N, which can be related to the rapid decrease in pH. The NH_3_-N concentration consistently increased during the 150 days ensiling. However, in the homLAB, hetLAB and MIX inoculated silages, most of the increase NH_3_-N concentration occurred during the first 3 days (**Figure 2C**), which correspond to the decrease in pH (**Figure 2A**). After the establishment of stable pH there were minimal increases in NH_3_-N. The increase in NH_3_-N in the early stages of ensiling may be the result of excessive fermentation and proteolytic activity by microorganisms and plant respiration. A decrease in pH inhibits the growth of undesirable bacteria and microbes, halts the production of NH_3_-N, and preserves the forage material for an extended period of time (Muck et al., 2018).

All the tested inoculants significantly improved the count of LAB and DM recovery, and decreased the count of yeast and mold of whole crop corn ensiled for 150 days. Notably, the greatest numbers of viable LAB were observed for homLAB, which improved the in-silo fermentation. Whereas, the lowest number of yeasts, mold and highest DM recovery was observed for hetLAB. The hetLAB decreased yeasts count by 60%, mold by 90% and increase DM recovery by 4.53%. In agreement with our findings *L. buchneri* as most used heterofermentative LAB strain can produce acetic acid to inhibit the growth of yeast and mold, thus improving DM recovery and aerobic silage stability (Guan et al., 2020). The LAB count decreased 72 hour exposure to air, and the maximum decrease (38%) was observed for homLAB, followed by control (23%), and the minimum for hetLAB (12%). In general the yeast count increased after 72 h exposure to air, but the magnitude of increase differed due to the type of inoculation. The maximum increase of 54% was observed for homLAB, followed by control (47%), while the hetLAB had the lowest increase of 27%. The greater spoilage in homLAB after exposure is because of the lower concentration of acetate, which is strongly antifungal, and greater concentration of lactate, which is a growth substrate for spoilage yeasts and mold (Oliveira et al., 2017). The highest aerobic stability of >72 h was recorded for hetLAB and MIX inoculated corn silages and lowest for control. It has been well established that *L. buchneri* increases the concentration acetic acid in silage, which inhibit the growth of yeasts and molds. Yeast is responsible for initiation of aerobic spoilage (Oliveira et al., 2017; Guan et al., 2020). Notably, like hetLAB, the MIX inoculant had lowest count of yeast, mold and highest DM recovery and aerobic stability, which could be related to the increase in acetic acid production as discussed before. Although, in the current study the positive effects of MIX did not exceed, the het LAB, it showed potential to improve aerobic stability and DM recovery.

## 5 Conclusions

The results of this study revealed that homLAB significantly improved silage fermentation quality and nutritional value, whereas hetLAB significantly improved DM recovery and aerobic stability of whole crop corn silage under hot summer conditions of the tropics. Among the additives, **t**he homLAB inoculated silages had the highest (*P* < 0.05) content of lactic acids (9.20% DM), soluble carbohydrates (3.53% DM), and lowest contents of acetic acid (1.16% DM), NH_3_-N (3.46% N) and pH (3.66), demonstrating desirable in silo fermentation. On the other hand, the hetLAB inoculated silages had the greatest (*P* < 0.05) content of acetic acids (2.01% DM), DM recovery (97.3%), and aerobic stability (>72 h). The greatest numbers of viable LAB were recorded in homLAB (8.13 log cfu/g) and MIX (7.89 log cfu/g) inoculated silages, while the lowest for control (6.29 log cfu/g). The lowest yeast (1.48 log cfu/g) and mold (0.22 log cfu/g) were recorded for hetLAB inoculated silage. Notably, like homLAB, inoculation with MIX inoculant significantly improved the rate and extent of pH decline and lactic acid production. On the other hand, hetLAB the MIX inoculant had lowest (*P* < 0.05) count of yeast, mold and highest DM recovery and aerobic stability. Although, in the current study the positive effects of MIX did not exceed, the individual (hom/het LAB) inoculants, it shows potential to improve in-silo fermentation and aerobic stability at the same time and needs further investigation, particularly with respect of dose rate.

## Data availability statement

The raw data supporting the conclusions of this article will be made available by the authors, without undue reservation.

## Ethics statement

The use and care of cannulated animals were approved by Institutional Animal Care and Use Committee of The University of Agriculture (Peshawar, KP, Pakistan).

## Author contributions

NAK: Conceptualization, Formal analysis, Methodology, Writing – original draft. NK: Investigation, Methodology, Resources, Writing – review & editing. ST: Conceptualization, Data curation, Formal analysis, Project administration, Supervision, Writing – review & editing. ZT: Conceptualization, Data curation, Funding acquisition, Investigation, Validation, Writing – review & editing.
